# Systematic review of clinical practice guidelines for long-term breast cancer survivorship: assessment of quality and evidence-based recommendations

**DOI:** 10.1038/s41416-025-03059-5

**Published:** 2025-05-17

**Authors:** Gustavo Adolfo Pimentel-Parra, Cristina García-Vivar, Paula Escalada-Hernández, Leticia San Martín-Rodríguez, Nelia Soto-Ruiz

**Affiliations:** Department of Health Sciences, Public University of Navarre and Navarra Institute for Health Research, Pamplona, Spain

**Keywords:** Quality of life, Breast cancer

## Abstract

**Background:**

Breast cancer is the most common cancer among women, with improved survival rates due to advances in early diagnosis and therapies. However, long-term survivors (≥5 years post-treatment, disease-free) face persistent physical, psychological, and social challenges requiring tailored, evidence-based care. Despite the growing survivor population, no systematic evaluation of Clinical Practice Guidelines (CPGs) for this group has been conducted. This study assesses the quality of CPGs and their evidence-based recommendations.

**Methods:**

A systematic review was conducted in PubMed, CINAHL, and Cochrane Library (2015–2023), including guidelines from major oncology organisations. The AGREE II instrument evaluated CPG quality across six domains, and recommendations were classified using a Primary Care survivorship framework: prevention, surveillance, care coordination, and long-term effect management.

**Results:**

Ten CPGs met inclusion criteria, with 7 classified as high quality. Most recommendations focused on prevention (adjuvant therapy, alcohol) and surveillance (follow-up, mammography), while gaps remained in lifestyle guidance, psychosocial support, and management of complications (lymphedema, osteoporosis, cognitive dysfunction). Care coordination and psychosocial interventions were inconsistently addressed.

**Conclusions:**

Current CPGs inadequately cover the complex needs of long-term survivors, particularly in psychosocial care. Evidence-based, patient-centred guidelines are urgently needed to optimise long-term outcomes and quality of life.

## Introduction

Breast cancer is a global public health challenge and one of the main causes of premature mortality in women worldwide [1]. In 2022, 2.3 million new cases of breast cancer were estimated, representing 11.6% of all cancer diagnoses and establishing breast cancer as the most common type of cancer among women [1]. In addition, ~666,000 deaths in 2022 were attributable to this disease (6.9% of all cancer deaths), highlighting its devastating impact worldwide [1]. Advancements in early diagnosis strategies, targeted therapies and multidisciplinary care have led to a significant increase in survival rates [2–4].

Globally, the 5-year relative survival rate for patients with breast cancer is 82%, reflecting significant disparities in access to health services between the most developed regions and those with limited resources [5]. The survival rate is 91% in the United States [6]; 92% in Australia [7]; ~83% in Europe, depending on the country [5, 8]. This progress has transformed breast cancer from a disease with high mortality to a chronic condition for many patients, with profound implications for their long-term quality of life (QoL) [9].

Breast cancer survivors face complex challenges that transcend the mere absence of active disease. According to the report *From Cancer Patient to Cancer Survivor: Lost in Transition*, cancer survival is divided into three stages: acute survivorship, which encompasses the period of diagnosis and initial treatment; extended survivorship, which includes the phase after treatment during which frequent surveillance is maintained to detect recurrences; and permanent survivorship or long-term survivorship, which begins 5 years after primary treatment when the risk of relapse decreases but the physical, psychological and social sequelae of cancer persist [10–12].

Long-term breast cancer survivors experience physical symptoms such as fatigue, chronic pain, lymphedema and sleep disturbances, as well as psychological problems such as anxiety and depression, which negatively impact their QoL and require specialised care [13–15]. In addition, these women require follow-up and long-term care strategies, including surveillance to monitor recurrence, management of late effects of previous therapies, and support for coping with psychosocial problems related to social, work and family reintegration [14–16]. The transition to long-term survivorship, typically marked at 5 years post-treatment, represents a critical shift in care [11, 17]. At this stage, oncologic follow-up often becomes less frequent, and many patients transition to primary or survivorship-focused care [10]. As a result, healthcare providers must address the evolving needs of survivors, including the management of late-onset treatment toxicities, the emergence of new comorbidities, and ongoing psychosocial challenges.

However, the complexity of long-term survivorship care requires a personalised approach, as follow-up needs vary depending on multiple factors, including age at diagnosis, tumour stage, molecular subtype (hormone receptor and HER2 status), type of treatment received, social determinants, and healthcare setting (community vs. specialised cancer centre) [9, 18, 19].

Additionally, differences in recurrence patterns among tumour subtypes significantly impact follow-up strategies [20]. While hormone receptor-positive (HR+) breast cancer can recur decades after diagnosis, HR-negative tumours have a very low recurrence rate beyond 8 years [21, 22]. This distinction reinforces the need for long-term survivorship care strategies tailored to individual recurrence risks, treatment history, and ongoing health concerns.

In this context, Clinical Practice Guidelines (CPGs) are essential tools to guide evidence-based healthcare, providing recommendations on diagnosis, treatment and follow-up care [23]. The CPGs support healthcare professionals in making health decisions and are useful for evidence-based clinical practice [24].In oncology, the CPGs have been shown to improve clinical outcomes and optimise patient management [25, 26]. However, the development of these guidelines is influenced by differences in methodology, evidence quality, and the evaluation criteria used by the various organisations that publish the CPGs. These variations can generate inconsistent recommendations, making it difficult to make clinical decisions and implement standardised care strategies [27].

Despite the growing interest in the care of long-term breast cancer survivors and the importance of CPGs for the clinical management of these women, to date, no systematic evaluation for the analysis of the quality of the CPGs available for this specific population has been performed. By specifically assessing CPGs for survivors 5 or more years post-treatment, this review aims to provide insights into whether current guidelines adequately address the unique needs of this population, beyond the extended survivorship phase. Therefore, the aim of this systematic review was to assess the quality of the CPGs for long-term breast cancer survivors and to identify evidence-based recommendations. This analysis aims to provide a comprehensive perspective to guide the implementation of targeted clinical practices to meet the unique needs of long-term breast cancer survivors effectively.

## Methods

### Research questions

What is the quality of the CPGs available to address long-term breast cancer survival? What recommendations do these CPGs provide for follow-up at 5 years after primary cancer treatment?

### Study design

A systematic review of CPGs and recommendations on long-term breast cancer survival was conducted to assess guideline quality and evidence-based clinical recommendations.

This review was designed and conducted in accordance with the recommendations for systematic reviews of CPGs [28] and the Preferred Reporting Items for Systematic Reviews and Meta-Analyses (PRISMA) statement [29, 30]. The protocol of this study was registered in the International Prospective Registry of Systematic Reviews (reference number, PROSPERO: CRD42023479083).

### Search strategy

Literature searches were conducted in PubMed, CINAHL, and Cochrane Library from January 1, 2015, to November 5, 2023, based on recommendations to update CPGs every 3–5 years [31, 32]. The strategy focused on three key concepts: breast cancer, CPGs, and survival, combining category searches with MeSH terms and keywords in titles and abstracts. Boolean operators ‘OR’ and ‘AND’ facilitated intra- and intercategory searches. No language restrictions were applied. Detailed search strategies for each database are in Appendix A (Tables S1–S3). Additionally, searches were conducted in leading oncology institutions and scientific societies, including: the Agency for Health care Research and Quality (AHRQ), American Cancer Society (ACS), American Society of Clinical Oncology (ASCO), Cancer Research UK, European Organization for Research and Treatment of Cancer (EORTC), European Society for Medical Oncology (ESMO), International Agency for Research on Cancer (IARC), Livestrong Foundation, National Cancer Institute (NCI), National Comprehensive Cancer Network (NCCN), National Institute for Health and Clinical Excellence (NICE), Spanish Society of Medical Oncology (SEOM), NCI Office of Cancer Survivorship (OCS), and World Cancer Research Fund (WCRF).

### Eligibility criteria

The inclusion and exclusion criteria are shown in Table 1. For this review, a CPG was defined as a set of recommendations aimed at improving patient health that are developed through a process that evaluates the available evidence in the literature [28] and is endorsed by government agencies, institutions or scientific societies [33]. In this review, long-term survivorship was defined as the stage that begins 5 years or more after diagnosis and primary treatment has been completed [11, 17].

**Table 1:** Eligibility criteria.

Inclusion criteria	Exclusion criteria
- CPG published between 2015 and 2023 that included recommendations for women older than 18 years who are breast cancer survivors in the long survival phase 5 years after completing active treatment. - Published in any language - Full text available^a^	- CPG published before 2015 - Older versions of the most recently updated CPG - Any literature that is not a CPG (for example, review articles, primary studies, book chapters, consensus statements, etc.) - Male population - Child and adolescent population - Palliative care population - Population with other types of cancer - Patients in the diagnosis or treatment phase (acute and extended survival)

^a^Guidelines without full-text access were excluded despite all efforts to retrieve them, and due to a lack of funding for this project, we were unable to purchase CPGs.

### Study selection

The records identified in the searches were exported to the online platform Covidence [34], and an initial screening of the titles and abstracts was performed, followed by a full-text screening on the basis of the predetermined eligibility criteria. This screening process was performed independently by two reviewers (GPP, NSR), and disagreements were resolved by a third reviewer (CGV).

### Quality assessment

Two reviewers (GPP, NSR) assessed CPG quality using the Appraisal of Guidelines for Research and Assessment (AGREE II) tool, which consists of 23 items across six domains: scope and objective (three items), participation (three items), rigour in preparation (eight items), clarity of presentation (three items), applicability (four items) and editorial independence (two items). Items were rated from 1 (Strongly disagree) to 7 (Strongly agree) [23, 35]. Each reviewer independently graded all 23 items.

The total scores for each domain (Standardized Domain Score [SDS]) were calculated using the following formula provided by the AGREE II tool [23, 35]:

$${PED}=\frac{{{{\rm{Score}}}}\; {{{\rm{obtained}}}}-{{{\rm{Minimum}}}}\; {{{\rm{possible}}}}\; {{{\rm{score}}}}}{{{{\rm{Maximum}}}}\; {{{\rm{possible}}}}\; {{{\rm{score}}}}-{{{\rm{Minimum}}}}\; {{{\rm{possible}}}}\; {{{\rm{score}}}}}\times100$$

$${{{\rm{Maximum}}}}\; {{{\rm{possible}}}}\; {{{\rm{score}}}}=7\left({{{\rm{strongly}}}}\; {{{\rm{agree}}}}\right)\times {{{\rm{No}}}}.\left({{{\rm{items}}}}\right)\times 2\left({{{\rm{evaluators}}}}\right).$$

$${{{\rm{Minimum}}}}\; {{{\rm{possible}}}}\; {{{\rm{score}}}}=1\left({{{\rm{strongly}}}}\; {{{\rm{disagree}}}}\right)\times {{{\rm{No}}}}.\left({{{\rm{items}}}}\right)\times 2\left({{{\rm{evaluators}}}}\right).$$

$${{{\rm{Score}}}}\; {{{\rm{obtained}}}}={{{\rm{The}}}}\; {{{\rm{sum}}}}\; {{{\rm{of}}}}\; {{{\rm{the}}}}\; {{{\rm{scores}}}}\; {{{\rm{obtained}}}}\; {{{\rm{from}}}}\; {{{\rm{each}}}}\; {{{\rm{evaluator}}}}\; {{{\rm{for}}}}\; {{{\rm{each}}}}\; {{{\rm{item}}}}.$$

According to the evidence [36, 37], CPGs scoring ≥60% in five or six domains were classified as high quality, those scoring ≥60% in three or four domains as moderate quality, and those with two or fewer as low quality. The overall assessment was based on the AGREE II instrument’s six domains, with a maximum score of 161 points (100%). Each evaluator independently assigned scores based on established criteria.

To determine relative CPG scores, the percentage was calculated by dividing the individual score by 161 and multiplying by 100. This percentage was then converted to the AGREE II 7-point scale using proportional calculation. For instance, a score of 100/161 equals 62.11%, which corresponds to 4.34 on the 7-point scale. This standardised approach ensures comparability and consistency in interpretation.

### Data extraction

The extraction of data from each CPG was carried out independently by two reviewers (GPP, NSR). Disagreements were resolved by consensus. The Covidence platform [34] was used to extract data, including characteristics of the publication (author, year of publication, title, journal, country or region of publication, developing organisation or institution, funding sources), CPG development methods and recommendations.

### Data analysis

The recommendations of the CPGs were classified using the clinical framework *A Framework for Comprehensive Breast Cancer Survivorship Care in the Primary Care Setting* [38], which is based on the report *From Cancer Patient to Cancer Survivors: Lost in Transition* [10], widely used in research and clinical practice related to cancer survival [39–42].

This comprehensive framework [38] establishes four essential domains for the primary care of cancer survivors: 1—prevention of recurrent and new cancers and late effects; 2—surveillance for recurrent or new cancers and assessment of physical and psychosocial late effects; 3—coordination between primary care providers and specialists to address all healthcare needs; and 4—intervention for long-term effects of cancer grouped by treatment, including surgery, chemotherapy, targeted therapy, radiation therapy, hormonal therapy, and psychosocial therapy (Table 2).

**Table 2:** Breast cancer survivorship care framework by Luctkar-Flude et al. [38].

Prevention of recurrent and new cancers and late effects	Surveillance for recurrent or new cancers and assessment of physical and psychosocial late effects	Coordination between primary care providers and specialists to address all healthcare needs	Intervention for long-term effects of cancer (grouped by treatment)
• Exercise • Nutrition • Weight management • Alcohol consumption • Immunisations • Smoking cessation • Sun exposure • Adjuvant therapy^a^	• History and physical • Mammography • Breast self-exam • Pelvic exam • Non-routine test	• Survivorship care plan • Specialist referrals	• Surgery: pain, lymphedema • Chemotherapy: peripheral neuropathy, cardiovascular complications, cognitive dysfunction, fertility, pregnancy and contraception, fatigue, menopausal symptoms • Targeted therapy: cardiovascular complications • Radiation therapy: cardiovascular complications, lymphedema, fatigue • Hormonal therapy: menopausal symptoms, fertility, pregnancy and contraception, osteoporosis, arthralgias and myalgias • Psychosocial issues: distress, anxiety and depression, sexual dysfunction, family and work

^a^Item added by the authors of this review.

In addition, a new category of recommendations was added by the authors to the prevention domain, called ‘adjuvant treatment’, to include recommendations that are related to this type of treatment but could not be classified into other categories. Adjuvant therapy refers to hormonal endocrine therapy used for patients with hormone receptor-positive (ER+/PR+) breast cancer. Adjuvant therapy includes medications such as tamoxifen or aromatase inhibitors (AIs) such as letrozole, anastrozole, or exemestane.

## Results

### Search results

**Fig. 1:**
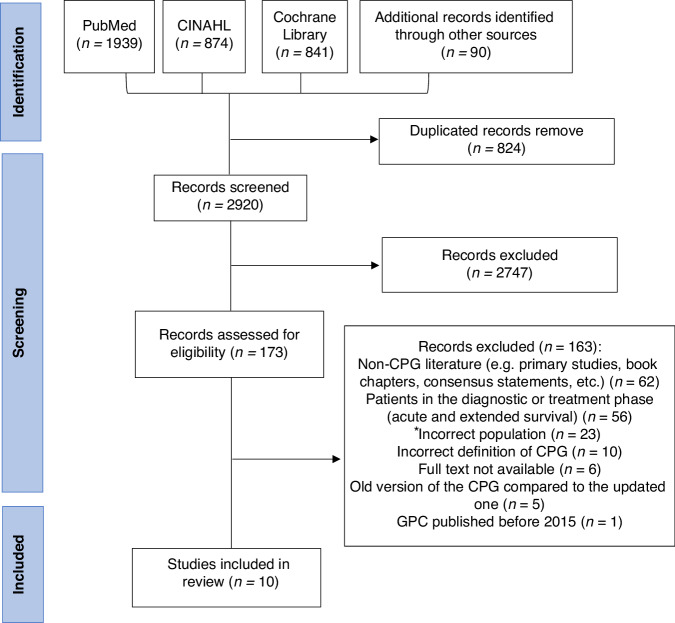
PRISMA flowchart of the study search and selection process. ^*^Incorrect population: Palliative care population (*n* = 13), Population with other types of cancer (*n* = 8), Child and adolescent population (*n* = 1), Male population (*n* = 1).

A total of 3744 records were identified through the bibliographic search. After removing 824 duplicates and excluding 2747 based on title and abstract screening, 173 full-text records were examined. Of these, 10 met the eligibility criteria (Fig. 1). Most exclusions were due to the article not being a CPG (62 records) or not addressing long-term cancer survivors (56 records).

### Characteristics of CPGs

The CPGs included in this review were published between 2016 and 2024 (Table 3). Two of these guidelines were from the United States [16, 43], two from Italy [44, 45] and one from each of the following countries or regions: Europe [46], Spain [47], Germany [48], the United Kingdom [49], Canada [50] and the Pan-Asian region [51].

**Table 3:** Characteristics of the included GPCs.

Source (Author)	Guideline title	Journal (Q:JIF)	Country or region	Development organisation or institution	Funds or sources of financing	Methods for guideline development				
						Specialists involved (developers)	Systematic literature review	Expert consensus	External expert review panel	Internal expert review panel
Burstein et al. [43]	Adjuvant Endocrine Therapy for Women With Hormone Receptor–Positive Breast Cancer: ASCO Clinical Practice Guideline Focused Update	Journal of Clinical Oncology (Q1:42,1)	United States	ASCO	ASCO	Medical oncologists, community oncologists, patient representation, methodologists	X	X	X	X
Runowicz et al. [16]	American Cancer Society/American Society of Clinical Oncology Breast Cancer Survivorship Care Guideline	CA: A Cancer Journal for Clinicians (Q1:503,1)	United States	ACS, ASCO	Centers for Disease Control and Prevention	Primary care physicians, medical oncologists, gynaecologists, surgical oncologists, radiation oncologists, cancer survivors, nurses	X	X	X	X
CCA [50]	Follow-Up Care for Early-Stage Breast Cancer: Clinical Practice Guideline BR-013—Version 3	NA: Not Applicable	Canada	CCA - GURU	CCA	Surgical oncologists, radiation oncologists, medical oncologists, nurses, pathologists, pharmacists, methodologists, patient representatives from across Alberta	X	X	X	X
Biganzoli et al. [44]	Breast Neoplasms Guidelines	NA: Not Applicable	Italy	AIOM	AIOM	Medical oncologist, methodologist, patient representatives	X		X	X
NICE [49]	Early and locally advanced breast cancer: diagnosis and management	NA: Not Applicable	United Kingdom	NICE	NICE	GP partners, general practitioners, breast surgeons, lay members, clinical nurse specialists, breast pathologists, clinical oncologists	X	X	X	X
Loibl et al. [46]	Early breast cancer: ESMO Clinical Practice Guideline for diagnosis, treatment and follow-up	Annals of Oncology (Q1:56,7)	Europe	ESMO	ESMO	Medical oncologists, surgical oncologists, radiation oncology specialists	X	X	X	X
Stangl et al. [48]	Evidence-based Guideline for the Early Detection, Diagnosis, Treatment and Follow-up of Breast Cancer	NA: Not Applicable	Germany	AWMF, DKG, DKH, DGGG	DKH	Oncologists, gynaecologists, radiologists, psycho-oncologists, geneticists, pathologists, geriatricians, obstetricians, mastologists—senologists, physiotherapists, rehabilitation specialists, surgeons, oncology and paediatric nurses, users, patients	X	X	X	X
Ciabattoni et al. [45]	AIRO Breast Cancer Group Best Clinical Practice 2022 Update	Tumori Journal (Q3:2,0)	Italy	AIRO Breast Cancer Group	LILT	Specialists in oncological radiotherapy	X		X	
Ayala de la Peña et al. [47]	SEOM-GEICAM-SOLTI clinical guidelines for early-stage breast cancer (2022)	Clinical and Translational Oncology (Q2:2,8)	Spain	SEOM, GEICAM, SOLTI	CRUE-CSIC agreement with Springer Nature	Oncologists who are experts in the treatment of breast cancer	X	X	X	
Park et al. [51]	Pan-Asian adapted ESMO Clinical Practice Guidelines for the management of patients with early breast cancer: a KSMO-ESMO initiative endorsed by CSCO, ISMPO, JSMO, MOS, SSO and TOS	Annals of Oncology (Q1:56,7)	Pan-Asian (Korea, China, India, Japan, Malaysia, Singapore, Taiwan)	KSMO, ESMO, CSCO, ISMPO, JSMO, MOS, SSO, TOS	ESMO	Medical oncologists, haematology-oncology specialists, gastrointestinal oncology specialists, breast surgeons, radiation oncologists, breast radiologists	X	X	X	X

*ACS* American Cancer Society, *AIOM* Associazione Italiana di Oncologia Medica, *AIRO* Italian Association of Radiation and Clinical Oncology, *AWMF* Association of Scientific Medical Societies in Germany, *CCA* Cancer Care Alberta, *CSCO* Chinese Society of Clinical Oncology, *DGGG* German Society for Gynecology and Obstetrics, *DKG* German Cancer Society, *DKH* German Cancer Aid, *ESMO* European Society for Medical Oncology, *GEICAM* Spanish Breast Cancer Research Group, *GURU* Guideline Resource Unit, *ISMPO* Indian Society of Medical and Pediatric Oncology, *JSMO* Japanese Society of Medical Oncology, *KSMO* Korean Society of Medical Oncology, *LILT* Lega Italiana per la Lotta ai Tumori, *MOS* Malaysian Oncological Society, *NICE* National Institute for Health and Care Excellence, *SEOM* Spanish Society of Medical Oncology, *SOLTI* Spanish Collaborative Group for the Study, Treatment and Other Experimental Strategies in Solid Tumors, *SSO* Singapore Society of Oncology, *TOS* Taiwan Oncology Society.

Of the 10 CPGs included, six were published in scientific journals. Four CPGs [16, 43, 46, 51] were published in high-impact journals (Q1) with a journal impact factor (JIF) between 503.1 and 42.1 (Table 3), and two CPGs [45, 47] were published in moderate-impact journals (Q2 and Q3). The other four CPGs [44, 48–50] were not published in scientific journals but were available as institutional reports published on official institutional websites.

The CPGs were developed by multidisciplinary teams, including oncologists, surgeons, radiation oncologists, gynaecologists, pathologists, pharmacists, physiotherapists, specialised nurses, geneticists, psycho-oncologists, geriatricians and methodologists (Table 3). Additionally, four CPGs [43, 44, 48, 50] included patient representatives.

Moreover, the recommendations included in these CPGs are primarily intended for oncologists, primary care practitioners, survivorship care providers, specialised nurses, psychologists, and other healthcare professionals involved in the long-term management of breast cancer survivors. Some recommendations may also be relevant for patients and caregivers to promote shared decision-making and self-management strategies.

All the teams adopted a systematic review-based approach and used external panels of experts for the review as part of their development methodology. Two CPGs [45, 47] did not include an internal review panel of experts, and two other CPGs [44, 45] did not use an expert consensus in their methodological process.

### Quality appraisal of the CPGs

Seven of the 10 CPGs [16, 43, 44, 46, 48–50] were classified as high quality, with scores greater than 60% in five or more domains and overall scores between 67% and 92% (Table 4). The three remaining guidelines [45, 47, 51] were classified as moderate quality, with scores greater than 60% in only three or four domains, with overall scores with the AGREE II tool varying between 50% and 67% (Table 4). Most of the guidelines included in this study had scores greater than 60% for the six domains included in the AGREE II tool (Table 4).

**Table 4:** AGREE II domain score of included clinical practice guidelines.

GPC ID	Domains (%)							Overall Quality
	Scope and purpose	Stakeholder involvement	Rigour of development	Clarity and presentation	Applicability	Editorial independence	Overall Assessment	
Runowicz et al. [16]	94	100	95	94	71	100	92	*High quality*
Biganzoli et al. [44]	61	69	86	89	63	100	67	*High quality*
Burstein et al. [43]	100	100	99	83	42	96	83	*High quality*
Stangl et al. [48]	92	100	92	94	29	100	75	*High quality*
CCA [50]	100	94	97	86	38	100	75	*High quality*
Loibl et al. [46]	81	69	61	89	25	96	67	*High quality*
NICE [49]	92	69	68	92	52	100	75	*High quality*
Park et al. [51]	94	22	50	97	6	96	67	*Moderate quality*
Ciabattoni et al. [45]	92	64	72	92	10	46	67	*Moderate quality*
Ayala de la Peña et al. [47]	94	47	43	97	4	96	50	*Moderate quality*

### Clinical recommendations for the care of long-term survivors of breast cancer by domain

The following clinical recommendations are specifically intended for breast cancer survivors who are more than 5 years post-treatment, have completed active treatment and are disease-free.

### Recommendations for prevention of recurrent and new cancers and late effects

The recommendations in the prevention domain were related only to alcohol consumption [48] and the new category of adjuvant treatment [16, 43, 44, 46–51] (Table 5). No recommendations were found in the CPGs for other aspects relevant to long-term survival, such as exercise, vaccines, nutrition, weight control, sun exposure or smoking cessation (Table 5).

**Table 5:** Classification of the clinical recommendations extracted from the CPGs included in the review.

Domains/Categories		Source: Author GPC included									
		Burstein et al. [43]	Runowicz et al. [16]	CCA [50]	Biganzoli et al. [44]	NICE [49]	Loibl et al. [46]	Stangl et al. [48]	Ciabattoni et al. [45]	Ayala de la Peña et al. [47]	Park et al. [51]
Prevention	Exercise—Inmunization—Nutrition—Weight management—Sun exposure—Smoking cessation										
	Alcohol consumption							X			
	Adjuvant treatment	X	X	X	X	X	X	X		X	X
Surveillance	Breast self-exam—Pelvic exam—Cholesterol and lipid screening										
	Non-routine tests							X			
	Mammography			X	X	X		X	X		
	History and physical		X	X	X		X	X	X	X	X
Coordination	Specialist referrals										
	Survivorship care plan				X			X			
Intervention	Pain—Lymphedema—Osteoporosis—Peripheral neuropathy—Cognitive dysfunction—Fertility, pregnancy & contraception—Menopausal symptoms—Fatigue—Arthralgias & myalgias										
	Cardiovascular complications				X						
	Distress, anxiety & depression						X				X
	Family & work						X				X
	Sexual dysfunction						X				X

Only the CPG by Stangl et al. [48] addressed the consumption of alcohol as an important factor in the prevention of recurrence in long-term survivors of breast cancer, including a recommendation to avoid the daily consumption of >12 g of pure alcohol in women with hormone-dependent breast cancer (Table 6).

**Table 6:** Recommendations from the clinical domains in CPGs for long-term breast cancer survivors.

Clinical domain: PREVENTION					
Categories within the domain	Recommendation^a^			Level of evidence	Author
Alcohol consumption	To avoid later relapses (>5 years after initial diagnosis), patients with receptor-positive disease should avoid a daily alcohol consumption of >12 g pure alcohol.			2A	Stangl et al. [48]
Adjuvant treatment	Endocrine therapy (tamoxifen, aromatase inhibitors, or ovarian suppression therapy) used as adjuvant systemic therapy for 5–10 years reduces the risk of recurrence and of subsequent second primary breast cancers and improves overall survival.			2A	Runowicz et al. [16]
	The oncology team may request re-referral to cancer centre after 2–5 years for discussion of potential switch in endocrine therapy (i.e., tamoxifen to an aromatase inhibitor) and/or for discussion of extended endocrine therapy (beyond 5 years). The request for re-referral will be indicated in the note from oncologist or delegate at the time of transition.			5B	CCA [50]
	After 5 years of tamoxifen, the continuation of tamoxifen for a further 5 years may be considered in women with resected infiltrating breast cancer with ER-positive and/or PgR-positive who are still premenopausal or perimenopausal, based on the results of ATLAS and aTToM studies; however, the benefit/harm ratio and the risk of recurrence for the individual patient must be assessed.			Not reported. Clinical Recommendation: Weak Positive	Biganzoli et al. [44]
	In postmenopausal women with resected ER-positive and/or PgR-positive infiltrating breast cancer who have completed 5 years of adjuvant tamoxifen, the use of aromatase inhibitors for 5 years should be considered, after evaluation of the benefit/risk ratio.			Not reported. Clinical Recommendation: Strong Positive	
	In postmenopausal women with resected ER-positive and/or PgR-positive infiltrating breast cancer, the extension of aromatase inhibitor therapy after the fifth year may be considered, after an accurate risk/benefit assessment.			Not reported	
	In women who are premenopausal when diagnosed with infiltrating breast cancer, are treated with tamoxifen for 5 years, and enter menopause during the adjuvant treatment with chemotherapy or tamoxifen, treatment with letrozole after 5 years of tamoxifen could be considered, assessing the benefit/harm ratio and the risk of recurrence for the individual patient.				
	There are no data supporting the continuation of hormone therapy beyond the fifth year in premenopausal patients treated with 5 years of ovarian suppression + tamoxifen or exemestane. In these patients, continuation of hormone therapy with tamoxifen or an AI may be considered based on the risk/benefit ratio and after confirmation of menopausal status. In patients who are candidates for hormone therapy with an AI, a complete evaluation of the menopausal status with repeated FSH and estradiol testing is necessary to confirm their postmenopausal status as accurately as possible.				
	In women with contraindications to aromatase inhibitors or who develop severe toxicities (e.g. musculoskeletal), tamoxifen for 5 years, or tamoxifen for 2–3 years followed by an aromatase agent for 2–3 years, may be considered.				
	In elderly patients in good overall condition, who have completed 5 years of treatment with tamoxifen, the use of anti-aromatase agents with an extended strategy may be considered.				
	Offer extended endocrine therapy (past the 5-year point) with an aromatase inhibitor for postmenopausal women with ER-positive invasive breast cancer who are at medium or high risk of disease recurrence and who have been taking tamoxifen for 2 to 5 years. Medium or high risk may include people who have lymph node-positive breast cancer, with tumours that are T2 or greater and higher grade.			Not reported	NICE [49]
	Consider extended endocrine therapy (past the 5-year point) with an aromatase inhibitor for postmenopausal women with ER-positive invasive breast cancer who are at low risk of disease recurrence and who have been taking tamoxifen for 2–5 years. Low risk may include people with lymph node-negative breast cancer, with smaller or lower-grade tumours.				
	Consider extending the duration of tamoxifen therapy for longer than 5 years for people with ER-positive invasive breast cancer.				
	Extended ET beyond 5 years should be considered in high-risk EBC; 7–8 years’ treatment duration seems sufficient for most patients at high risk.			1A	Loibl et al. [46]
	In postmenopausal women, AIs, used either upfront or sequentially after 2–3 years of tamoxifen, offer lower risk of recurrence compared with tamoxifen alone, especially in higher-stage cancers. Standard treatment duration is 5 years but extended durations to 7 or 10 years further lower recurrence risk and increase survival, particularly in higher-stage cancers.			Not reported	
	The use of aromatase inhibitors, either as upfront therapy after 2–3 years of tamoxifen, or as extended adjuvant therapy after 5 years of tamoxifen, has shown promise in postmenopausal women with hormone-positive tumours.			Not reported	Stangl et al. [48]
	After 5 years of tamoxifen, the indication for extended endocrine therapy shall be evaluated for each patient with ER+ breast cancer. Indications should be based on the weighing of the risk of relapse and the therapy-associated side effects (toxicity, reduced adherence). The current menopausal status of the patient shall be taken into account when choosing endocrine therapy.			1A	
	Premenopausal patients shall be treated with tamoxifen for at least 5 years. Antiestrogenic therapy with tamoxifen 20 mg per day shall be carried out over a period of 5–10 years or until relapse, depending on the risk of relapse. The indication for extended therapy depends on the risk of recurrence and the patient’s wishes.				
	Patients with hormone receptor-positive breast cancer shall receive adjuvant endocrine therapy with tamoxifen, usually for 5 years. No data are available for treatment beyond 5 years. As with female breast carcinoma, this may be considered in individual cases.			Expert Consensus (EC): Strong Consensus	
	Extended adjuvant therapy (optimal duration: 7.5–8 years) should be discussed with nearly all patients, except those with a very low risk of relapse.			1A	Ayala de la Peña et al. [47]
	Extended adjuvant ET with tamoxifen for up to 10 years should be considered in high-risk patients who remain premenopausal during the entire adjuvant period.			1B	
	AI after 5 years of tamoxifen (letrozole and anastrozole) as extended adjuvant therapy, especially in intermediate- to high-risk (node-positive) patients.			1A	
	Extended adjuvant therapy with AI for more than 8 years offers minimal benefit.			1C	
	In high-risk postmenopausal patients who decline or do not tolerate AI, 10 years of tamoxifen should be considered.			1A	
	For premenopausal women, tamoxifen for 5–10 years is a standard of care.			1A	Park et al. [51]
	AIs can be used upfront (non-steroidal AI and exemestane), after 2–3 years of tamoxifen (non-steroidal AI and exemestane) or as extended adjuvant therapy, after 5 years of tamoxifen (letrozole and anastrozole).			1A	
	There is only a minimal benefit for the use of AIs for more than 5 years.			1C	
	Many women with node-negative breast cancer are potential candidates for and may be offered extended AI therapy for up to a total of 10 years of adjuvant endocrine treatment based on considerations of recurrence risk using established prognostic factors. However, as the recurrence risk is lower, the benefits are likely narrower for such patients. Women with low-risk node-negative tumours should not routinely be offered extended therapy.			Not reported	Burstein et al. [43]
	Women with node-positive breast cancer should be offered extended AI therapy for up to a total of 10 years of adjuvant endocrine treatment.				
	Women who receive extended adjuvant endocrine therapy should receive no more than 10 years of total treatment.				
	Preferred options for extended therapy include an AI for up to a total of 10 years or a sequence of tamoxifen for 2–3 years followed by 7–8 years of an AI or a sequence of tamoxifen for 5 years followed by an AI for 5 years.				
	10 years of tamoxifen therapy is recommended for premenopausal or postmenopausal women who have not tolerated or prefer not to take AI-based treatment.				
**Clinical domain: SURVEILLANCE**					
History and physical		It is recommended that primary care clinicians (a) should individualise clinical follow-up care provided to breast cancer survivors based on age, specific diagnosis, and treatment protocol and as recommended by the treating oncology team and should make sure the patient receives a detailed cancer-related history and physical examination every 3–6 months for the first 3 years after primary therapy, every 6–12 months for the next 2 years, and annually thereafter.		2A	Runowicz et al. [16]
		History and physical examination of the breast(s), chest wall ±reconstructed breast, and supraclavicular and axillary nodes. Also consider assessing arms for lymphedema, especially if axillary nodal dissection or regional nodal irradiation. Frequency: every 6 months for 2 years, then annually.		2A and 5A	CCA [50]
		Patient history and objective examination should be obtained every 3–6 months in the first 3 years after the primary treatment, every 6–12 months in the following 2 years and then annually.		Not reported	Biganzoli et al. [44]
		Regular follow-up visits are recommended every 6 months from years 4–5 and annually thereafter. The interval of visits can be adapted to the risk of relapse and patient needs.		5A	Loibl et al. [46]
		Follow-up examinations should be carried out half-yearly in the 5th year after local primary therapy and annually from the 6th year onwards.		Expert Consensus (EC): Strong Consensus	Stangl et al. [48]
		History and physical examination: Every 3–6 months for the first 3 years after primary treatment, and then every 6–12 months until reaching 5 years. Annually after 5 years from primary treatment in the absence of symptoms.		Not reported	Ciabattoni et al. [45]
		For early breast cancer, regular follow-up visits are recommended every 6 months from years 3–5, and annually thereafter.		3A	Ayala de la Peña et al. [47]
		Regular follow-up visits are recommended every 6–8 months from years 3–5 and annually thereafter. The interval of visits should be adapted to the risk of relapse and patients’ needs.		5A	Park et al. [51]
Mammography		Mammography: post-treatment mammogram of intact breast(s) 1 year after diagnostic mammogram (or 6+ months post-definitive radiotherapy), then annually; performed at an accredited mammography centre. Mammography of an entirely reconstructed breast (autologous or implant) is not recommended as there is no significant residual natural breast tissue to image. Routine breast cancer screening with breast MRI is not indicated.		5C	CCA [50]
		Mammography should be performed within 1 year of the mammogram that allowed to diagnose the tumour (in women undergoing conservative surgery, a mammogram at least 6 months after the end of radiotherapy), then once a year.		Not reported	Biganzoli et al. [44]
		Offer annual mammography for 5 years to all people who have had or are being treated for breast cancer, including DCIS. For women, continue annual mammography past 5 years until they enter the NHS Breast Screening Programme (NHSBSP) in England or the Breast Test Wales Screening Programme (BTWSP) in Wales.		Not reported	NICE [49]
		Follow-up examinations for breast cancer after breast-conserving therapy (BCT) or mastectomy in normal as well as higher risk patients: Ipsilateral breast (BCT): annual mammography and mammary sonography are recommended starting from the fourth year. Mastectomy: annual sonography are recommended starting from the fourth year. contralateral breast: annual mammography and sonography if necessary are recommended starting from the fourth year.		Expert Consensus (EC): Strong Consensus	Stangl et al. [48]
		If the risk of relapse is low, after 10 years of follow-up, the X-ray frequency of mammography can be extended to 2 years.			
		Mammographic controls even after 10 years in women undergoing breast cancer surgery with a reasonable life expectancy (even in elderly women) to reduce the risk of mortality.		Not reported	Ciabattoni et al. [45]
Non-routine tests		During the 5th year and thereafter, Laboratory examinations, examinations using imaging techniques (exception: mammography and mammary sonography) should be performed only in case of clinical suspicion of recurrence and/or metastases.		Expert Consensus (EC): Strong Consensus	Stangl et al. [48]
**Clinical domain: COORDINATION**					
Survivorship care plan			Based on the available evidence, women who have undergone chest radiotherapy (CRT) before the age of 30 with a cumulative dose ≥10 Gy should be invited to participate in a specific surveillance programme from the age of 25 or at least 8 years after CRT, including the following assessments: - Annual bilateral CE-MRI with the same protocol used to screen women at high risk for hereditary/family factors. - Annual bilateral mammography or tomosynthesis with 2D reconstruction. Mammography and MRI can be performed simultaneously or alternately every 6 months. Upon reaching the age for invitation to organised screening programmes, the woman’s risk profile should be reassessed and discussed to decide for annual or biennial mammography-based screening (possibly with tomosynthesis) or continuation of annual mammography and MRI.	Not reported	Biganzoli et al. [44]
			Follow-up management: Breast cancer must be considered a chronic condition, even in patients who remain disease-free, and therefore must receive the care and attention reserved for all other medical chronic conditions. At the end of the follow-up by the oncology specialist (usually after 5 years), the patient can be referred to her General Practitioner (GP), with indication to receive annual mammographic checks and clinical examinations.		
			A follow-up period of at least 10 years is necessary because of the tumour biology of breast cancer. Therapy monitoring must be continued for at least 10 years.	Not reported	Stangl et al. [48]
**Clinical domain: INTERVENTION**					
Cardiovascular complications			The guidelines of the European Society of Oncology suggest, in the absence of specific indications, a cardiological evaluation 6 months after the end of chemotherapy, to be repeated annually for 2 or 3 years and then every 3–5 years. Patients at high risk, those who have received high cumulative doses of anthracyclines and elderly patients may be monitored more frequently.	Not reported	Biganzoli et al. [44]
Distress, anxiety & depression			Long-term survivorship problems including psychological needs and issues related to work, family and sexuality should be addressed.	5A	Loibl et al. [46], Park et al. [51]
Sexual dysfunction					
Family & work					

*ET* endocrine therapy, *EBC* early breast cancer, *AI* aromatase inhibitor, *DCIS* ductal carcinoma in situ, *MRI* magnetic resonance imaging.

^a^The CPG recommendations have been transcribed verbatim to ensure fidelity to the published guidelines, considering the limited number of CPGs in the study context.

The analysis of the CPG recommendations revealed both similarities and discrepancies in the therapeutic strategies for adjuvant treatment, particularly with respect to the use and duration of endocrine therapy, personalisation of the treatment and risk assessment. Five CPGs recommended the use of tamoxifen in premenopausal women and AIs either as the initial treatment or sequentially after tamoxifen in postmenopausal women, for at least 5 years after treatment [44, 46–48, 51]. However, the recommendations differ in the extent of treatment beyond 5 years (Table 6). Some guidelines suggest prolonging tamoxifen use for up to 10 years in premenopausal women or women who do not tolerate Ais [16, 43, 47, 50, 51] and extending the use of AIs in high-risk postmenopausal women up to a maximum of 10 years [16, 43, 44, 46, 48–50]. In contrast, some authors [47, 51] have been reluctant to recommend a prolonged duration of endocrine therapy, indicating that the benefits beyond 7–8 years are minimal.

### Recommendations for surveillance for recurrent or new cancers and assessment of late physical and psychosocial effects

In the surveillance domain, the recommendations were related to medical history and physical examination [16, 44–48, 50, 51], mammographies [44, 45, 48–50] and non-routine tests [48] (Table 5). No recommendations related to breast self-examination, pelvic examination, or cholesterol and lipid screening were found.

In the CPGs included in this review, the recommendations regarding the frequency of clinical follow-up in long-term breast cancer survivors vary. In general, the recommendations agree on an annual follow-up frequency beginning in the sixth year after primary treatment (Table 6). However, during the fifth year, significant variations are observed: four of the CPGs [46–48, 51] recommend a clinical follow-up every 6 months, and three CPGs [16, 44, 45] extend this interval, recommending a frequency of every six to 12 months. On the other hand, the Canadian Cancer Society CPG [50] differentiates itself by extending the frequency of follow-up to 12 months beginning in the fifth year.

With respect to mammography as follow-up, all but one [45] of the CPGs reviewed recommend annual mammography beginning in the fifth year (Table 6). All the guidelines emphasise the importance of individualising mammographic follow-up strategies according to factors such as the characteristics of the tumour, the type of breast surgery (conservative or mastectomy), age, and the risk of relapse or mortality.

Finally, one of the CPGs [48] recommended laboratory tests and imaging studies (except mammography and breast ultrasound) beginning in the fifth year in cases of clinical suspicion of recurrence and/or metastasis (Table 6).

### Recommendations for coordination between primary care providers and specialists

In the coordination domain, recommendations regarding survival care plans were identified [44, 46], whereas no references included recommendations on referrals to specialists (Table 5).

Two of the 10 CPGs [44, 48] agreed on the need for long-term follow-up (5–10 years or more), with an emphasis on regular evaluation. However, Biganzoli et al. [44] propose early transfer to primary care, whereas Stangl et al. [48] highlight the importance of continuous monitoring due to the chronic nature of the disease (Table 6). Both guidelines underscore the importance of establishing clear protocols for the transition between levels of care, ensuring continuity of care.

### Recommendations for interventions for long-term effects of cancer

For the intervention domain, recommendations concerning cardiovascular complications [44], psychological needs and problems related to work, family and sexuality [46, 51] were included (Table 5). However, no recommendations were identified in the CPGs for the following categories: pain, lymphedema, osteoporosis, peripheral neuropathy, cognitive dysfunction, fertility, pregnancy and contraception, menopausal symptoms, fatigue, arthralgia and myalgia.

Regarding cardiovascular complications, Biganzoli et al. [44] recommend evaluations every 3–5 years in long-term breast cancer survivors and more frequent monitoring in those at high risk, such as patients treated with high cumulative doses of anthracyclines or the elderly (Table 6).

The guidelines of Park et al. [51] and Loibl et al. [46] are the only ones that recommend the inclusion of psychosocial needs and work-related issues, family and sexuality as a complement to the recommendations for medical follow-up, highlighting the importance of multidimensional care beyond only monitoring recurrence or complications (Table 6).

## Discussion

This systematic review is the first to identify, compare and evaluate the quality of CPGs with recommendations specifically directed at survivors in the long-term survival period, that is, more than 5 years after primary cancer treatment. Therefore, this review provides an overview of the clinical recommendations currently available for this population, allowing an understanding of the existing guidelines and their applicability in the context of long-term survival.

### Absence of CPGs for long-term survivors of breast cancer

The main finding of this review was the absence of CPGs specifically designed for this population group. The guidelines predominantly focus on the acute and extended phases of survival (diagnosis, treatment, and the short post-treatment phase, up to 5 years), leaving the unique and persistent needs of long-term survivors unaddressed (permanent survival, + 5 years).

Only 10 CPGs for breast cancer patients included any recommendation for this stage of survival (Table 5). Considering that CPGs are essential tools to guide healthcare, support clinical decision-making, and promote evidence-based practice [23, 24], the scarcity of recommendations for long-term breast cancer survivors highlights important unmet care needs and the feeling of helplessness that many of these survivors experience once their cancer treatments are completed [52, 53]. However, it is important to acknowledge that, although CPGs are typically updated every 3–5 years, many are not revised within this timeframe and may not incorporate the latest findings or recommendations for survivors beyond 5 years after the completion of primary treatment.

After an intense period of treatment and frequent check-ups, many survivors feel ‘lost in transition’ and face a difficult period marked by uncertainty and a lack of clear guidelines for their long-term care [10]. Upon reaching the five-year mark, many patients are discharged from specialised care into the care of primary care professionals. However, this transition is often accompanied by significant barriers, such as a lack of specific information or communication by oncologists, which leaves primary care professionals without data to guide the care of survivors [54–56]. In addition, these professionals face challenges such as limited knowledge about survival management, the absence of clear guidelines, lack of time, and, in some cases, the lack of confidence of the patients who might feel safer under the care of specialists [54, 55]. These factors create gaps in the continuity and quality of care for long-term survivors of breast cancer [57].

The identified recommendations were classified using the *Framework for Comprehensive Breast Cancer Survivorship Care in the Primary Care Setting* by Luctkar-Flude et al. [38], which establishes four essential domains for the care of cancer survivors. The absence of specific recommendations contrasts with the principles established in this framework [38] and the report *From Cancer Patient to Cancer Survivors: Lost in Transition* [10]. Both publications highlight the importance of comprehensive care that encompasses prevention, surveillance, clinical management, coordination and psychosocial aspects, highlighting the need to generate more inclusive guidelines adapted to long-term survivors.

### Prevention and health promotion in long-term survivors of breast cancer

The few specific recommendations for long-term survivors were mostly classified in the prevention and surveillance domains, specifically in the three categories of ‘adjuvant treatment’, ‘history and physical’ and ‘mammography’.

In the prevention domain, more than 96% of the recommendations focus on strategies related to adjuvant treatment. All CPGs highlight the relevance of this therapeutic approach to reduce the risk of cancer recurrence and improve survival rates by addressing possible residual tumour cells after primary treatment [16, 43, 44, 46, 48–51]. There is a general consensus among CPGs regarding the extension and personalisation of adjuvant treatment, with the aim of optimising its clinical efficacy while minimising potential adverse effects [16, 43, 44, 46–51]. However, there are discrepancies even within the same CPG, where the extension of treatment up to 10 years is recommended, but the limited efficacy beyond 7–8 years is simultaneously noted [47, 51]. These inconsistencies underscore the need for greater clarity and uniformity in the recommendations to avoid confusion in their clinical application.

Regarding alcohol consumption as a risk factor, only one CPG recommends limiting intake to less than 12 g/day in patients with receptor-positive breast cancer [48]. However, this recommendation contrasts with the emerging evidence that suggests a trend towards abstinence (0 g/day) as the safest standard, given the association between alcohol consumption, even in small amounts, and the increased risk of recurrence or appearance of new cancers [58–60]. This discrepancy highlights the need to update the recommendations related to alcohol in the CPGs aimed at this population.

However, other aspects of the prevention of recurrence or appearance of new cancers, the management of side effects and the promotion of a healthy lifestyle, such as nutrition, physical exercise, weight control, cessation of smoking or sun exposure, did not receive any attention in these CPGs. This omission underscores a significant gap in evidence-based guidance for comprehensive prevention and person-centred care beyond drug treatment.

Compared with patients in the acute and extended survival stages [16, 44, 46, 49] and those with other chronic diseases, such as diabetes [61], for which clear prevention guidelines related to physical exercise, nutrition and other key aspects of self-care have been established, specific recommendations for long-term cancer survivors remain limited and unstructured. Although there are some general guidelines that address these aspects, they tend to lack specificity in terms of the survival period to which they are directed [62, 63]. In addition, these recommendations frequently lack the methodological rigour of evidence-based CPGs. This discrepancy may be related to two factors: first, the risk of relapse is greater in the early stages of survival, and second, the long-term follow-up of participants in most studies extends only up to 5 years after diagnosis [64].

The absence of CPGs developed through standardised methodological processes makes it difficult for health professionals to implement consistent guidelines [24]. This lack represents a significant barrier to addressing the specific needs of prevention and care in this population, highlighting the urgency of developing evidence-based tools that enable uniform and effective care in this critical stage of survival [65].

The ‘history and physical’ and ‘mammography’ categories in the surveillance domain reported by most CPGs emphasise the importance of medical visits and/or physical examinations and mammographic controls to detect possible risk factors, sequelae or relapses. In general, the CPGs have similar recommendations regarding the frequency of annual clinical follow-up beginning in the sixth year and annual mammographic controls beginning in the fifth year. This uniformity indicates a consensus on the importance of regular surveillance for the early detection of recurrence or new breast cancers in long-term survivors.

However, other important surveillance tests for the detection of new or recurrent cancers, as well as the evaluation of physical and psychosocial late effects, are omitted [38, 39]. Only one CPG [48] recommended the importance of performing laboratory tests and imaging tests. However, the types of tests or parameters that should be evaluated are not specified, which indicates a lack of clarity in the recommendations concerning other types of surveillance tests aimed at long-term survivors of breast cancer.

### Need to promote care beyond cancer

The coordination and intervention domains were the most neglected domains in the CPGs, with only three [44, 48] and two [44, 46, 51] recommendations, respectively. With respect to the coordination domain, the CPG of Biganzoli et al. [44] states that ‘Breast cancer must be considered a chronic condition’, highlighting the importance of continuous monitoring, consistent with what is observed in other chronic diseases. Similarly, Stangl et al. [48] emphasise that the follow-up period for breast cancer should be extended to at least 10 years, given the complexity of its tumour biology. This perspective is consistent with current trends that view breast cancer as a chronic disease comparable to high blood pressure or diabetes [66, 67]. This consideration lies in the fact that breast cancer is not completely resolved with primary treatment, and both the late effects of treatments and the sequelae of the disease tend to persist in the long term, significantly impacting the patients’ QoL [66, 67].

The intervention domain included recommendations on cardiovascular complications, psychological needs, work-related issues, family, and sexuality, but in a general and superficial way. While long-term breast cancer survivors face significant physical and psychosocial challenges—such as pain, lymphedema, osteoporosis, cognitive impairment, sexual health issues, stress, anxiety, depression, and job reintegration difficulties [14, 38]—these needs are not well represented in CPGs. Although some guidelines recommend interventions for these issues, they mainly focus on acute and extended survival phases (≤5 years post-treatment) [16, 44, 50, 68], highlighting a lack of guidance for long-term survivors. For instance, Davies et al. [68] recommend monitoring lymphedema every 3 months in the first year post-surgery and then every 2 years until 5 years. Similarly, the Canadian Cancer Society CPG [50] advises bisphosphonate use for up to 5 years in women at high risk of recurrence or treatment-related bone loss. However, none address follow-up beyond 5 years, despite survivors reporting ongoing needs.

The lack of clarity in recommendations and absence of specific guidelines for this survival stage hinder comprehensive care, underscoring the urgent need to develop precise guidelines to support long-term survivors [10, 38, 39]. This gap may stem from limitations in the applicability domain, which scored lowest among all domains, indicating inadequate attention to implementation barriers, strategies, and resource impact [23, 35].

### Strengths and limitations

A rigorous process was followed to identify relevant CPGs for long-term breast cancer survivors. This study presents several methodological strengths, reinforcing its robustness. First, the AGREE II instrument, a widely validated tool, assessed the methodological quality of the included CPGs. Additionally, data extraction and evaluation underwent peer review via Covidence, ensuring transparency, reproducibility, and minimising bias.

Another key strength is the review’s breadth and depth. By synthesising CPGs from various professional and governmental organisations, it provides a comprehensive perspective on evidence-based recommendations. This review not only identified best practices but also analysed key differences between sources, offering a clearer picture of discrepancies and areas of convergence in current recommendations.

It is crucial to address the challenge of identifying specific recommendations for long-term survivors (over 5 years post-treatment). The CPGs fail to clearly differentiate survival stages, making it difficult to extract tailored recommendations for those who have completed active treatment. This limitation may stem from only four CPGs [43, 44, 48, 50] including patient representatives in their development. The lack of patient involvement likely contributes to the absence of recommendations addressing the unique needs of breast cancer survivors in permanent survival, highlighting a gap in patient-centred care.

### Implications and future directions

The limited focus on long-term survivors highlights a critical gap in care. This underscores the need for specific recommendations addressing their unique needs, including QoL interventions, tailored prevention, and comprehensive psychosocial care. Integrating these into CPGs will enhance patient outcomes and promote holistic, equitable oncology practices. Additionally, transitioning from specialised to primary care without well-defined CPGs creates gaps in continuity. Strengthening communication between oncology and primary care teams is essential for effective survivor management.

Therefore, developing CPGs specifically for long-term breast cancer survivors is essential. These guidelines should provide clear recommendations on relapse prevention, follow-up protocols (frequency, objectives, etc.), late-effect management, psychological support, work reintegration, and social well-being beyond 5 years post-treatment. Their development should involve oncology specialists, primary care professionals, and long-term survivors to ensure continuity of care and address specific needs effectively. Policymakers must also prioritise long-term follow-up programmes by allocating resources, offering professional incentives, and implementing evaluation systems to ensure high-quality care for long-term cancer survivors.

## Conclusion

This review highlights a critical gap in the CPGs for long-term breast cancer survivors. The lack of specific recommendations, particularly in follow-up and psychosocial care, underscores the need for a paradigm shift in guideline development. Future CPGs should assume that personalised surveillance and treatment recommendations are provided by oncologists at the time of patient discharge to primary care or survivorship settings. Therefore, guidelines should focus on key aspects essential for long-term survivorship, including the early identification of recurrence, the management of late complications (e.g., osteopenia/osteoporosis, metabolic syndrome, lymphedema), and comprehensive psychosocial support. By integrating these priorities within established frameworks and robust evidence, guidelines can better address the evolving needs of this growing population while remaining applicable to healthcare providers in diverse settings.

## Supplementary information

Appendix A

## Data Availability

All data analysed in this study were obtained from publicly available clinical practice guidelines published by professional organisations or institutions. Full references and links to the included guidelines are provided in the article and/or supplementary material.
