# Supplementary material for: Systematic review of clinical practice guidelines for long-term breast cancer survivorship: assessment of quality and evidence-based recommendations
Source: Br J Cancer. 2025 May 17;133(2):178–93. doi: 10.1038/s41416-025-03059-5 (PMC12304102; doi:10.1038/s41416-025-03059-5)
Supplement: Supplementary file 1 — Appendix A [file 41416_2025_3059_MOESM1_ESM.docx]

# Appendix A

## **Table S1**. Search strategy used and number of studies found in PubMed (Medline).

| PubMed (n= 1939) | |
| --- | --- |
| #1 | Breast Neoplasms[MeSH] OR Breast Diseases[MeSH] OR "Breast Neoplasm*"[Tiab] OR "Breast Disease*"[Tiab] OR "Breast Cancer"[Tiab] OR "Breast Tumor*"[Tiab] OR "Breast Carcinoma*"[Tiab] OR "Cancer of Breast"[Tiab] OR "Cancer of the Breast"[Tiab] OR "Human Mammary Carcinoma*"[Tiab] OR "Mammary Carcinoma*"[Tiab] OR "DCIS"[Tiab] OR "LCIS"[Tiab] OR "Ductal Carcinoma* in Situ"[Tiab] OR "Lobular Carcinoma* in Situ"[Tiab] |
| #2 | Practice Guideline[Publication Type] OR Practice Guidelines as Topic[MeSH] OR Guideline[Publication Type] OR Guidelines as Topic[MeSH] OR Health Planning Guidelines[MeSH] OR "Guideline*"[Tiab] OR "Clinical Protocol*"[Tiab] OR "How-to Guide*"[Tiab] OR "Recommendation*"[Tiab] OR "Statement*"[Tiab] |
| #3 | Cancer Survivors[MeSH] OR Survivors[MeSH] OR Survivorship[MeSH] OR "Survivor*"[Tiab] OR "Follow-up*"[Tiab] OR "Post-treatment*"[Tiab] |
| #4 | #1 AND #2 AND #3 |
| Filters | • January 1, 2015 – November 5, 2023 |

## **Table S2**. Search strategy used and number of studies found in CINAHL (via EBSCOHost).

| CINAHL (n= 874) | |
| --- | --- |
| #1 | (MH "Breast Neoplasms+") OR (MH "Breast Diseases+") OR (TI ("Breast Neoplasm*" OR "Breast Disease*" OR "Breast Cancer" OR "Breast Tumor*" OR "Breast Carcinoma*" OR "Cancer of Breast" OR "Cancer of the Breast" OR "Human Mammary Carcinoma*" OR "Mammary Carcinoma*" OR "DCIS" OR "LCIS" OR "Ductal Carcinoma* in Situ" OR "Lobular Carcinoma* in Situ")) OR (AB ("Breast Neoplasm*" OR "Breast Disease*" OR "Breast Cancer" OR "Breast Tumor*" OR "Breast Carcinoma*" OR "Cancer of Breast" OR "Cancer of the Breast" OR "Human Mammary Carcinoma*" OR "Mammary Carcinoma*" OR "DCIS" OR "LCIS" OR "Ductal Carcinoma* in Situ" OR "Lobular Carcinoma* in Situ")) |
| #2 | (MH "Practice Guidelines") OR (TP "Practice Guidelines") OR (TI ("Guideline*" OR "Clinical Protocol*" OR "How-to Guide*" OR "Recommendation*" OR "Statement*")) OR (AB ("Guideline*" OR "Clinical Protocol*" OR "How-to Guide*" OR "Recommendation*" OR "Statement*")) |
| #3 | (MM "Cancer Survivors") OR (MM "Survivors") OR (MM "Survivorship") OR (TI ("Survivor*" OR "Follow-up*" OR "Post-treatment*")) OR (AB ("Survivor*" OR "Follow-up*" OR "Post-treatment*")) |
| #4 | #1 AND #2 AND #3 |
| Filters | • January 1, 2015 – November 5, 2023  • Apply related words  • Apply equivalent subjects  • Also search within the full text of articles |

## **Table S3**. Search strategy used and number of studies found in the Cochrane Central Register of Controlled Trials (CENTRAL) (via the Cochrane Library).

| Cochrane Library (CENTRAL) (n= 841) | |
| --- | --- |
| #1 | MeSH descriptor: [Breast Neoplasms] explode all trees |
| #2 | MeSH descriptor: [Breast Diseases] explode all trees |
| #3 | ("Breast Neoplasm" OR "Breast Neoplasms" OR "Breast Disease" OR "Breast Diseases" OR "Breast Cancer" OR "Breast Tumor" OR "Breast Tumors" OR "Breast Carcinoma" OR "Breast Carcinomas" OR "Cancer of Breast" OR "Cancer of the Breast" OR "Human Mammary Carcinoma" OR "Human Mammary Carcinomas" OR "Mammary Carcinoma" OR "Mammary Carcinomas" OR "DCIS" OR "LCIS" OR "Ductal Carcinoma in Situ" OR "Ductal Carcinomas in Situ" OR "Lobular Carcinoma in Situ" OR "Lobular Carcinomas in Situ"):ti,ab,kw |
| #4 | #1 OR #2 OR #3 |
| #5 | MeSH descriptor: [Practice Guideline] explode all trees |
| #6 | MeSH descriptor: [Practice Guidelines as Topic] explode all trees |
| #7 | MeSH descriptor: [Health Planning Guidelines] explode all trees |
| #8 | ("Guideline" OR "Guidelines" OR "Clinical Protocol" OR "Clinical Protocols" OR "How-to Guide" OR "How-to Guides" OR "How-to Guidebook" OR "Recommendation" OR "Recommendations" OR "Statement" OR "Statements"):ti,ab,kw |
| #9 | #5 OR #6 OR #7 OR #8 |
| #10 | MeSH descriptor: [Cancer Survivors] explode all trees |
| #11 | MeSH descriptor: [Survivors] explode all trees |
| #12 | MeSH descriptor: [Survivorship] explode all trees |
| #13 | ("Survivor" OR "Survivors" OR "Survivorship" OR "Follow-up" OR "Follow-ups" OR "Follow-up's" OR "Follow-upping" OR "Post-treatment" OR "Post-treatments"):ti,ab,kw |
| #14 | #10 OR #11 OR #12 OR #13 |
| #15 | #4 AND #9 AND #14 |
| Filters | • January 1, 2015 – November 5, 2023 |
| Search link | https://www.cochranelibrary.com/web/cochrane/advanced-search/search-manager?search=7322596 |
